# A case report of phosphaturic mesenchymal tumor-induced osteomalacia

**DOI:** 10.1097/MD.0000000000009470

**Published:** 2017-12-22

**Authors:** Weiqian Wu, Chongyang Wang, Jianwei Ruan, Feng Chen, Ningjun Li, Fanghu Chen

**Affiliations:** a Department of Orthopedics; b Department of Respiration; c Department of Pathology, Taizhou Municipal Hospital, Taizhou, Zhejiang, China.

**Keywords:** neoplastic osteomalacia, phosphate urinary mesenchymal tumor, radiotherapy, surgical resection

## Abstract

**Rationale:**

Tumor-induced osteomalacia (TIO) is a rare and often misdiagnosed syndrome. Surgical resection is currently the first line treatment for TIO.

**Patient concerns:**

Here we report the case of a 49-year-old woman presented with intermittent pain in the right chest and bilateral hip that had persisted for over two years.

**Diagnoses:**

She was diagnosed of TIO caused by a phosphaturic mesenchymal tumor based on the following examinations. Laboratory tests revealed high serum alkaline phosphatase, high urinary phosphorus, hypophosphatemia and normal serum calcium levels. 18-FDG PET/CT indicated a systemic multi-site symmetrical pseudo fracture and a tumor in the 7th right rib.

**Interventions:**

Curettage of the tumor was performed, and pathological analysis also confirmed our diagnoses as a phosphaturic mesenchymal tumor.

**Outcomes:**

At seven months post-surgery, the symptoms were relieved, proximal muscle strength was improved and serum levels of phosphorus and alkaline phosphatase normalized. The bilateral femoral neck and bilateral pubic bone fractures were blurred in the pelvic plain X-ray, suggesting that the fracture was healing.

**Lessons:**

This case report strengthened the importance of recognition of this rare disease to avoid delay of diagnosis and surgical removal of the causative tumor is recommended.

## 1 Introduction

Tumor-induced osteomalacia (TIO) is a rare paraneoplastic syndrome^[1]^ characterized by high serum levels of alkaline phosphatase, high urinary phosphorus levels, and multisite progressive bone pain with false fractures. TIO typically presents with normal levels of serum calcium, parathyroid hormone, decreased or normal 1,25 (OH)2-D3, increased alkaline phosphatase, hypophosphate, and high urinary phosphorus.^[2]^ TIO tumors can be divided into 4 subtypes: phosphaturic mesenchymal tumor-mixed connective tissue variant (PMTMCT), osteoblastoma-like variant, ossified fibroids-like variant, and nonossifying fibroma-like variant. The PMTMCT variant was the most common one, accounting for 70% to 80%. Common microscopic features of PMTMCT histomorphology include highly vascularized spindle cells, visible osteoclast-like multinucleated giant cells, bone-like metaplasia or cartilage-like structures, visible cell mucinous degeneration, calcification, and old bleeding. Since PMTMCT are rare and characterized by complex and nonspecific histological, pathologists have not reached a consensus on their systematic diagnosis. Due to the slow tumor growth and covert location, the rate of TIO misdiagnosis is high. Herein, we report a case of TIO caused by PMT. By providing detailed clinical data, we aim to improve clinicians’ understanding of TIO.

## 2 Case report

All procedures performed in this case study were performed in accordance with the ethical standards of the national research committee and with the 1964 Helsinki declaration and its later amendments or comparable ethical standards.

A 49-year-old woman in perimenopause presented with intermittent pain in the right chest and both hips that has persisted for over 2 years. She first sought care at a local hospital for right chest pain in October 2013, and no abnormalities were found on a chest CT. Oral pain medication (etoricoxib 120 mg PO QD) was given, but the pain did not ease. The patients returned to hospital with the band-like chest pain of unknown cause in May 2014. In August 2014, she returned to the hospital with bilateral hip and lower back pain. Pelvic plain X-ray indicated no obvious abnormalities, but the results of the lumbar MRI revealed compression fractures on the vertebral bodies of T12 and L1. Oral analgesics and antiosteoporosis drugs (calcitonin nasal spray 20 μg QD, calcium 600 mg PO QD, and calcitriol 0.25 μg PO QDqd po) were given. In 2016, a pelvic plain X-ray showed bilateral femoral neck fractures and a left suprapubic fracture. Antiosteoporosis treatment was given without joint replacement. In November 2016, the patient came to our hospital for treatment.

Physical examination revealed thoracic compression syndrome and bilateral groin midpoint tenderness, and bilateral hip joint activity was restricted. Limb muscle and physiological reflex were normal, and no pathological reflex was found.

Laboratory findings before surgery were as follows: high serum alkaline phosphatase (379 U/L), normal serum calcium (2.04 mmol/L), hypophosphatemia (0.8 mmol/L), low serum 25-(OH)-D (12.4 ng/mL), high serum parathyroid hormone (96.3 pg/mL), and ACTH and cortisol levels were normal. Sex hormones including FSH, progesterone, LH, PRL, E2, and testosterone levels were normal except estradiol, which was elevated (368 ng/mL). Serum levels of tumor markers, including CA-199, CA-125, CEA, AFP, and SCC, were normal. FGF-23 concentration and gene transcript analysis were unavailable.

**Figure 1:**
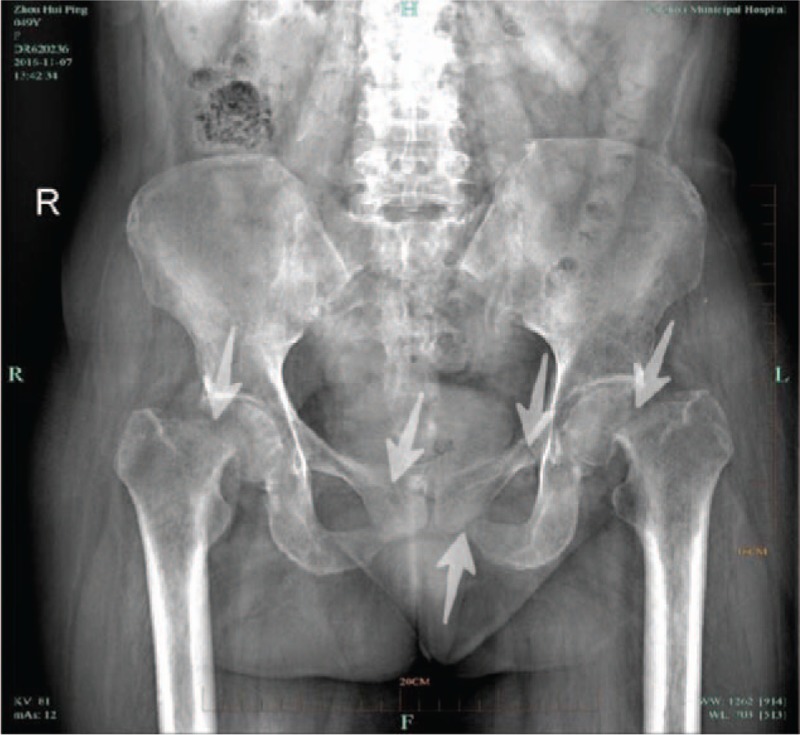
Preoperative pelvic X-ray suggested that bilateral femoral neck fracture and bilateral pubic fractures (indicated by white arrow).

**Figure 2:**
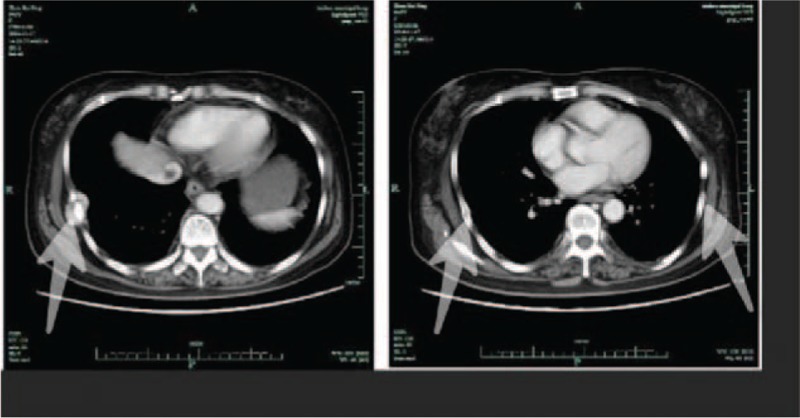
Chest computed tomography (CT) suggested multiple bilateral rib fractures (indicated by white arrow), destruction of the right side of the 7th, 8th rib bone, and a local soft tissue mass.

A radiograph pelvic plain X-ray taken in November 2016 (Fig. 1) indicated bilateral femoral neck fracture and bilateral pubic fractures. A chest CT (2016-11) (Fig. 2) revealed multiple bilateral rib fractures, destruction of the right side of the 7,8 bone, and a local soft tissue mass. PET/CT (18F-FDG) revealed multiple bilateral rib fractures, bilateral femoral neck fractures, bilateral pubic fractures, destruction of the 7th rib, and a soft tissue mass. Fractures were caused by osteomalacia as a result of the 7th rib tumor.

Detailed clinical characteristics of the patients were as follows: no positive family history and other genetic diseases, perimenopausal women with regular past menstrual cycles, and adult onset; the main clinical manifestation was bone pain at multiple. Lesions were observed to affect the ribs, femoral neck, and pubis progressively. Laboratory findings included hypoglycemia, high urinary phosphorus, high serum alkaline phosphatase, and parathyroid hormone. Serum calcium levels, cortisol levels and rhythm, sex hormone levels, and tumor markers were normal. Pelvic plain film and lung CT suggest a false cortical fracture; PET-CT revealed the location of the tumor, and hypophosphatemic osteomalacia was suggested to be the diagnosis at the 7th rib tumor. According to the above characteristics, a diagnosis of hypochondrogenic osteomalacia was made, but the cause of hypophosphatemic osteomalacia was unknown.

**Figure 3:**
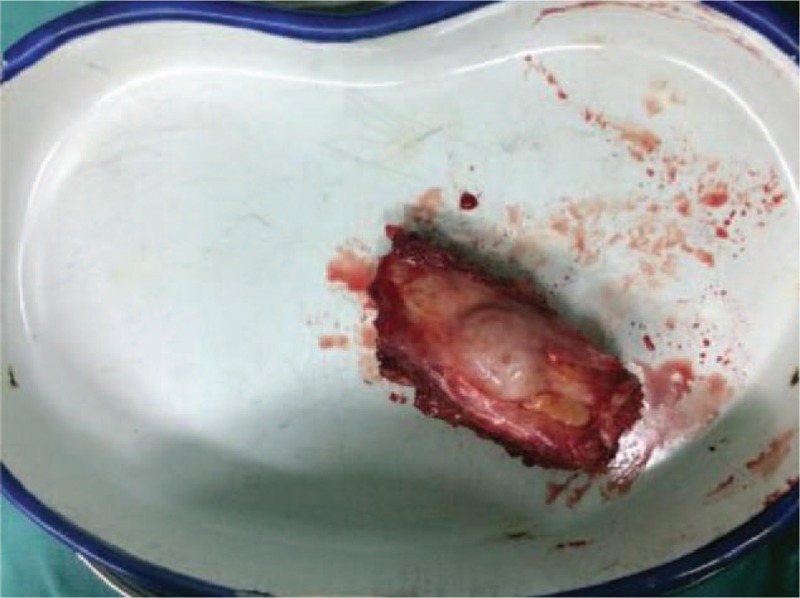
Tumor (2.1 × 3.2 cm) resection was performed.

**Figure 4:**
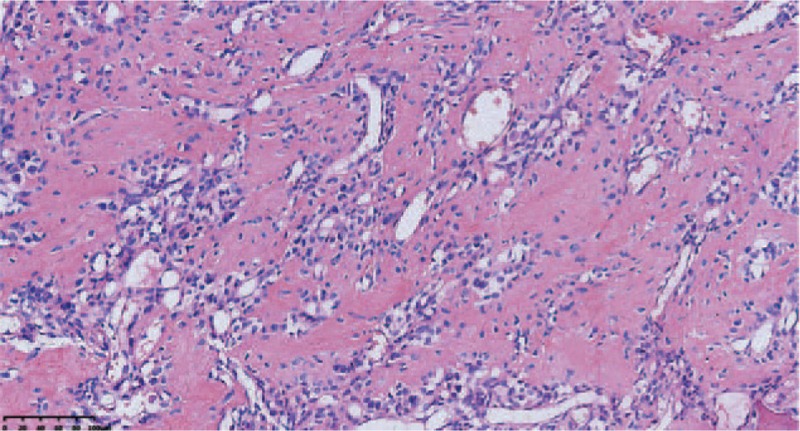
Pathology examination suggested highly vascularized spindle-cell tumor mixed with chondritic cells (magnification at 200×).

On December 12, 2016, rib tumor (Fig. 3) resection was performed under general anesthesia. Pathological analysis revealed highly vascularized spindle cells, accompanied by chondritic cells (Fig. 4). Immunohistochemical staining indicated that the tumor was Vim (+), CD68 (−), EMA (−), LCA (−), Syn (−), S100 (−), Ki67 (−), CD56 (+), CK (broad), and CgA (−). Combined with the clinical characteristics, these data suggested phosphaturic mesenchymal tumor.

**Figure 5:**
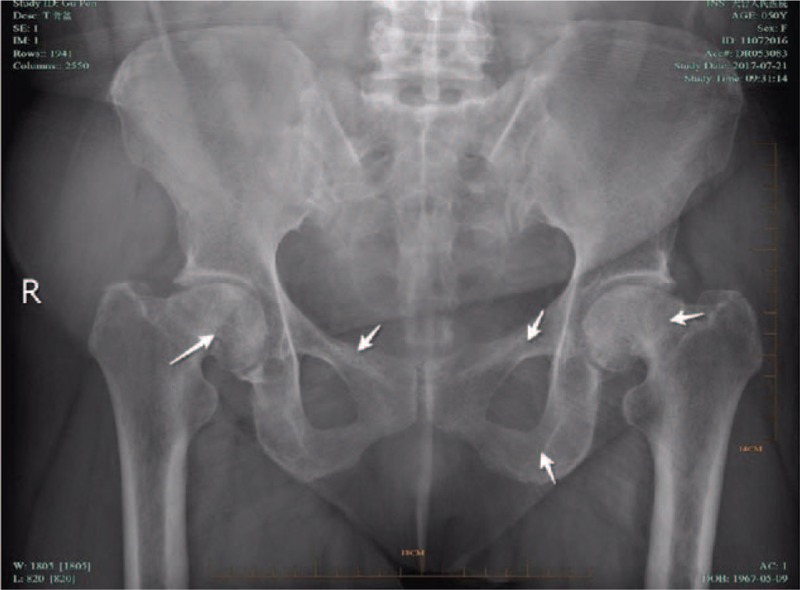
Postoperative pelvic X-ray at 7 months showed blurred fracture lines for both bilateral femoral neck and bilateral pubic fractures (indicated by white arrow).

After surgery, the patient's blood phosphorus was 0.93 mmol/L and alkaline phosphatase was 225 U/L. Seven months after surgery, symptoms were relieved and proximal muscle strength was improved. Both blood phosphorus (0.85 mmol/L) and alkaline phosphatase (120 U/L) levels normalized. Pelvic radiograph (Fig. 5) revealed blurring of the bilateral femoral neck and bilateral pubic fractures. Although genetic analysis was not performed and blood FGF-23 was not measured in this case, the patient's symptoms were significantly improved after surgery. Considering the characteristic of false fracture healing, the clinical diagnosis of TIO was clear.

## 3 Discussion

Previously reported causes of TIO include environmental factors such as lack of sunlight and fluoride exposure, poor diet, and drug use. TIO has also been reported secondary to liver and gastrointestinal diseases (Crohn disease and nontropical inflammatory bowel disease), endocrine disease (hyperthyroidism), chronic renal failure, tumor (osteomalacia, carcinogenic cartilage disease), acquired vancomini, and hereditary hypophosphatemia. In this case, the patient was physically handicapped and no exposure to the sunshine around the residence for unknown time. The patients had no family history of TIO or gastrointestinal, liver, or kidney diseases. Her menstrual cycle prior to menopause was regular; levels and rhythm of sex hormones and adrenaline did not suggest endocrine disease. She had normal skin with no “coffee milk spots” and no other symptoms such as neurofibromatosis were observed. The patient had normal blood calcium levels, but high serum parathyroid hormone levels, indicating a compensatory mechanism.

For patients with TIO, the clear positioning of the tumor and qualitative diagnosis of TIO is crucial for effective treatment. Most cases of TIO happen in the bone and soft tissue, most often the limbs. The slow growth and small size of tumors hinder detection.^[3]^ This may explain why the patient was not diagnosed within the first 2 years of symptomatic disease. Distinguishing between hypophosphatemia and genetically associated hypophosphatemia is important.^[4,5]^ As PMTMCT tumors are typically rich in blood flow, F-18FDG PET/CT is a very sensitive, yet nonspecific, diagnostic tool.^[6]^ High-resolution whole-body MRI imaging can effectively confirm the location of tumor.^[5,7]^ In addition, studies have shown that 68Ga-DOTATATE PET is a promising and diagnostic tool for locating TIO tumors.^[8–10]^

Haeusler et al^[11]^ suggested venous blood detection of FGF-23 was necessary to detect tumors. In some cases, FGF-23 concentration was measured in the venous blood at several sites to localize tumors.^[12–14]^ However, Houang et al^[15]^ reported that both FGF-23 and SSTR2A positive staining of PMTS is a highly sensitive measure of TIO, but is not specific to TIO. Nonetheless, dual negative staining can be used to exclude TIO test.

Complete and definitive surgical resection is the first choice treatment for TIO.^[4,16]^ Octreotide replacement therapy has also been shown to be useful for octreotide-positive patients.^[17,18]^ Phase I clinical trials indicated that anti-FGF-23 antibodies were effective and safe in adult patients with XLHR, and this therapy may be applicable for TIO patients in the future.^[19]^ Patients should also be routinely administered phosphate and vitamin D if the tumor location could not be defined. Although most cases of PMT are benign, PMT can be malignant.^[3,20]^ Radiotherapy is an option for tumors that could not be removed and are not completely resected.^[5,19]^ However, guidelines and dosing instructions are yet been established. The prognosis of TIO after tumor resection is good.^[16,17,21]^ Abnormal biochemical indicators typically return to normal after surgery, and clinical symptoms will ease within a few weeks of surgery.^[5]^

TIO is a rare clinical subtumor syndrome, and surgical resection is the first and the most effective treatment. Where complete removal of the tumor is not possible, radiotherapy is an important treatment to reduce recurrence or metastasis.
