# A case report of phosphaturic mesenchymal tumor-induced osteomalacia: Erratum

**DOI:** 10.1097/MD.0000000000009624

**Published:** 2018-01-12

In the article, “A case report of phosphaturic mesenchymal tumor-induced osteomalacia”,^[1]^ which appeared in Volume 96, Issue 22 of *Medicine*, there is a mistake in the Case report section and the sentence should have appeared as:

Oral analgesics and antiosteoporosis drugs (calcitonin nasal spray 20 mg QD, calcium 600 mg PO QD, and calcitriol 0.25 mg PO QD) were given.
